# Transplacental Transmission of *Plasmodium falciparum* in a Highly Malaria Endemic Area of Burkina Faso

**DOI:** 10.1155/2012/109705

**Published:** 2011-11-30

**Authors:** Alphonse Ouédraogo, Alfred B. Tiono, Amidou Diarra, Edith C. Christiane Bougouma, Issa Nébié, Amadou T. Konaté, Sodiomon B. Sirima

**Affiliations:** ^1^Centre National de Recherche et de Formation sur le Paludisme, 1487 Avenue de l'Oubritenga, BP 2208, Ouagadougou 01, Burkina Faso; ^2^Groupe de Recherche Action en Santé, BP 10248, Secteur 25, Somgandé, Rue 25.26, Porte 79, Ouagadougou 06, Burkina Faso

## Abstract

Malaria congenital infection constitutes a major risk in malaria endemic areas. In this study, we report the prevalence of transplacental malaria in Burkina Faso. In labour and delivery units, thick and thin blood films were made from maternal, placental, and umbilical cord blood to determine malaria infection. A total of 1,309 mother/baby pairs were recruited. Eighteen cord blood samples (1.4%) contained malaria parasites *(Plasmodium falciparum)*. Out of the 369 (28.2%) women with peripheral positive parasitemia, 211 (57.2%) had placental malaria and 14 (3.8%) had malaria parasites in their umbilical cord blood. The umbilical cord parasitemia levels were statistically associated with the presence of maternal peripheral parasitemia (OR = 9.24, *P* ≪ 0.001), placental parasitemia (OR = 10.74, *P* ≪ 0.001), high-density peripheral parasitemia (OR = 9.62, *P* ≪ 0.001), and high-density placental parasitemia (OR = 4.91, *P* = 0.03). In Burkina Faso, the mother-to-child transmission rate of malaria appears to be low.

## 1. Introduction

In malaria high-transmission areas, some population groups are at considerably higher risk of infection with *Plasmodium falciparum* and development of malaria morbidity or mortality than others. These include children less than five years of age and pregnant women. Malaria contributes significantly to perinatal disease burden in terms of pregnancy loss, prematurity due to preterm labor, and intrauterine growth retardation [1]. Malaria infection during pregnancy poses a substantial risk to the mother, her fetus, and the neonate. In areas of stable malaria transmission such as Burkina Faso, where adult women have considerable acquired immunity, *Plasmodium falciparum *infection during pregnancy does not cause symptomatic malaria, but may lead to maternal anemia as well as placental and cord blood malaria infection, especially among primigravidae and secundigravida [2–4]. 

Placental malaria is defined as the accumulation of *Plasmodium*-infected erythrocytes in the intervillous space in the placenta, causing histologic changes including leukocyte-induced damage to the trophoblastic basement membrane. The placental infection does not reflect the existence of peripheral infection over a short period preceding the delivery or whether it is related to infection during pregnancy. Susceptibility to this may be correlated to high exposure to malaria and repeated episodes of parasitemia during the pregnancy [5].

Vertical transmission of malaria from mother to foetus through the placenta and umbilical cord is defined as umbilical cord blood parasitemia. The transplacental transmission of *Plasmodium falciparum *from mother to fetus has long been well-described [6, 7]. 

The direct burden of neonatal malaria infection in terms of prevalence is not well-described in malaria endemic areas. In fact, the method used to identify congenital transmission is peripheral blood of newborns or umbilical cord blood. Malaria parasites have been detected only rarely in the peripheral blood of newborns, whether the blood specimen is collected at the time of birth or hours later [8, 9]. In studies in which both umbilical cord blood and infant peripheral blood were obtained at the time of birth, the parasite load in the babies' peripheral blood has always been lower than that in the umbilical cord blood [9–11].

Studies published so far have documented contradictory levels of this burden. In countries without endemic malaria, congenital malaria has occurred in children born to women who have immigrated from malarious areas. However, transplacental transmission of *Plasmodium falciparum *has been found to be rare in malaria-endemic areas, ranging from about 1 to 5% [12–15]. In contrast, data from recent studies on the burden of congenital and neonatal malaria, while scarce and contradictory, have indicated a high burden (more than 15%) in parts of sub-Saharan Africa [13, 15–18].

In Burkina Faso, the real prevalence of neonatal malaria is unknown but is estimated to be even higher. This assessment is based on presumptive malaria diagnosis. The present study was designed to determine the real burden of transplacental transmission, the risk factors associated with transplacental transmission, and the prevalence of cord blood and placental malaria parasitemia in malaria holoendemic area of Burkina Faso. 

These results represent a pooled analysis of studies on malaria prevention in pregnant women [4, 19, 20] examining umbilical cord blood to determine the frequency of transplacental transmission of *Plasmodium falciparum.*

## 2. Materials and Methods

### 2.1. Study Site

The first study took place within six delivery units (DUs) of the Koupela health district, which is located around 120 km east of Ouagadougou. The second study was conducted in one DU of the Health District of Bousse. Both study sites have been extensively described elsewhere [4, 19, 20]. Malaria transmission is stable, with marked seasonality in both sites. Transmission is intense during the rainy season (June to October). The main malaria vectors are *Anopheles gambiae, Anopheles arabiensis, *and *Anopheles funestus*. The annual entomological inoculation rates range from 10 to 500 infective bites per individual. *Plasmodium falciparum *is responsible for more than 90% of malaria infections. 

### 2.2. Study Population

The study participants were pregnant women who voluntarily consented to participate in trials of malaria prophylaxis during pregnancy. They were encouraged to deliver at the health facility where the study samples were collected for processing. 

### 2.3. Clinical Procedures

Women delivering at the health facility, after giving informed consent, were asked a standard series of questions focused on sociodemographic characteristics, history of fever, antimalarial drug use, and the use of antimalarial chemoprophylaxis and bednets. Capillary blood was obtained by fingerstick for malaria blood film preparation. Placental blood films were prepared by identifying the maternal side of the placenta, wiping away excess blood, cutting into the surface, and placing pooled blood onto a slide. Umbilical cord blood samples were obtained by wiping away excess blood from a clamped cord, piercing it with a lancet, and placing a drop of expressed blood on a slide.

A detailed clinical examination was done on each newborn infant within 24 hours of delivery. Neonates were weighted with an electronic digital scale (±10 grams) (Tanita Corporation, Tokyo, Japan). The Dubowitz scoring system was used to estimate gestational age, using findings from physical and neurologic examinations. Scoring by APGAR index was performed at delivery but was not recorded in this study.

### 2.4. Lab Methods

All blood films (maternal, placental, and cord) were stained with Giemsa and examined for parasites at the “Centre National de Recherche et de Formation sur le Paludisme” immunoparasitology laboratory in Ouagadougou. For thick films, parasites and leukocytes were counted in the same fields until 500 leukocytes were counted. Parasite densities were estimated using an assumed leukocyte count of 8,000 leukocytes/*μ*L. Parasite densities were calculated according to the following formula: number of parasites counted × 8000/number of leucocytes counted. Thin films were then used to determine species when thick films were positive. All slides were double-read by two independent microscopists. If the ratio of densities from the first two readings was greater than 1.5 or less than 0.67, or if fewer than 30 parasites were counted with a difference of more than 10 parasites between the two readings, the slide was evaluated a third time. The geometric mean of the parasite density of the two most concordant results of the three readings was taken as the final result. When the discordance was only in terms of positivity, the slide was also evaluated a third time and the definitive result was based on the majority verdict for positivity.

### 2.5. Definitions

Blood film results were considered to be positive if any asexual-stage parasites were identified and negative if no parasites were seen in 100 high-power fields. 

Prematurity was defined as a gestational age <37 weeks as estimated by Dubowitz examination.

We defined low birth weight as a birth weight of <2,500 g with a gestational age ≥37 weeks.

### 2.6. Statistical Analysis

Data collected in the study questionnaire were verified, then double-entered, and validated with EpiInfo, version 6.04 fr (Centers for Diseases Control and Prevention, Atlanta, USA). Data were analyzed using Epi-info and Stata 7.0. The analysis included data from births of all enrolled participants who delivered at the study centre clinic for whom data were available. Continuous, normally distributed data were described by mean and standard deviation and nonnormally distributed data by the median or geometrical mean and range. Proportions were compared using the Chi-square test and normally distributed continuous variables were compared using Student's *t*-test. Statistical results were considered significant, when the two-sided *P* value was <0.05.

### 2.7. Ethical Considerations

The study was discussed with health authorities and local leaders to obtain their assent. The study was reviewed and approved by the Burkina Faso Ministry of Health ethical committee. Informed consent (by signature or thumbprint) was obtained after the consent document was read to the women in the local language. For illiterate mothers, the informed consent discussion process was witnessed by an impartial individual.

## 3. Results

### 3.1. Characteristics of Women at the Delivery Unit

In total, 1,374 women delivered at the health unit; 1,309 women delivered singleton infants and provided data in the form of peripheral blood smear, placental blood, and umbilical cord blood. The profile of enrolled women is summarized in Table 1. Most of the women spoke Moore, a language indigenous to this region of Burkina Faso. The mean age (SD) of the women was 23.90 ± 5.89 years. The self-report indicated that ownership and use of insecticide-impregnated materials (bed nets and/or curtains) was reported by approximately one-third of the women. The rate of use of insecticide-impregnated materials (bed nets and/or curtains) was very low in the study population. All of the women used drug prophylaxis during their pregnancy (IPTp/SP or weekly prophylaxis with chloroquine).

Primigravidae comprised 35% of the total sample size, and primigravidae and secundigravida together made up 56% of the total sample size. The mean duration of gestation was 38.66 ± 3.89 weeks. The mode of delivery of babies was spontaneous for 97.5% of the mothers. 

Two women died during delivery as a consequence of eclampsia. Other complications during the delivery included peripartum haemorrhage, placental retention, and retroplacental haemorrhage. Fifty stillbirths (3.6%) and 18 (1.3%) miscarriages occurred at the DUs. 

The weight range of babies was 700 g to 4,700 g with a mean weight of 2,875 ± 450 g. Sixteen percent of the babies presented low birth weight at delivery (Table 1).

### 3.2. Maternal Peripheral Parasitemia: Placental and Umbilical Cord Blood Parasitemia

Over one-third of the women were parasitaemic. Indeed, of the 1,309 pregnant women, *Plasmodium falciparum* was detected in peripheral blood from 369 (28.2%) of the women at the time of delivery. Overall, the geometric mean of the maternal peripheral parasite density was 1,106.47 (95% CI 871.58–1404.67) parasites/*μ*L.  Table 2 shows the prevalence of peripheral, placental, and umbilical cord parasitemia. *Plasmodium falciparum *was detected in the placentas of 255 (19.5%) women; 28.5% of these belonged to primigravidae and 11.4% to multigravidae. The difference in infection between the primigravidae and the multigravidae was statistically significant (*P* < 0.0001). All of the women received malaria prophylaxis during pregnancy. 

Umbilical cord blood parasitemia with *Plasmodium falciparum *was detected in 18 (1.4%) babies, and the geometric mean umbilical cord blood parasitemia was 315.69 (95% CI 87.02–1145.20) parasites/*μ*L. Of these, 2.4% (11) belonged to primigravidae and 0.5% (3) to multigravidae. Of the 18 babies with *Plasmodium-falciparum*-positive umbilical cord blood parasitemia, 17 were positive for trophozoites and 1 was positive for schizonts only. Both schizonts and trophozoites were present in seven babies, and one newborn had a triple-positive association (trophozoite + schizont + gametocyte). Five newborns (27.8%) out of the 18 infected with *Plasmodium falciparum* had low birth weight. 

Out of 369 (28.2%) women with peripheral positive parasitemia, 211 (57.2%) had positive placental malaria and 14 (3.8%) had malaria parasites in the umbilical cord blood. Eleven (5.2%) of the 211 (57.2%) women with positive placental smears for malaria had malaria parasites in the umbilical cord blood. Of the 18 babies infected with *Plasmodium falciparum*, 14 (77.8%) were born from mothers with positive peripheral parasitemia. For two newborns with *Plasmodium falciparum* infection, no peripheral or placental infection was found in the mother. None of the babies with cord blood positive for malaria had fever within the first 24 h of life. 

The number of pregnant women with peripheral, placental, and umbilical infection and high peripheral parasitemia (>10,000 parasites/*μ*L) decreased with increasing gravidity (Table 2).

The combination of being born to a mother with both peripheral and placental infection conferred a greater likelihood of infection of the baby (61%). Being born with only maternal peripheral infection reduced the likelihood of having umbilical cord parasitemia (17%). There was an association between umbilical cord blood infection and maternal peripheral infection (*P* < 0.001). Being born with only placental infection reduced the rate of umbilical cord parasitemia (11%). There was also an association between umbilical cord blood infection and placental infection (*P* < 0.001) (Table 3). 

Being born to a mother with maternal peripheral parasite density ≥5,000 parasites/*μ*L or placental parasite density ≥10,000 parasites/*μ*L conferred a greater likelihood of umbilical cord parasitemia (Table 3).

### 3.3. Risk Factors

We further examined the correlation between risk factors and the presence or absence of umbilical cord parasitemia to determine possible factors that might affect infection with malaria parasites in umbilical cord blood. Univariate logistic regression analysis indicated that being born with maternal parasitemia, being born with maternal parasite density ≥ 5,000 parasites/*μ*L, being born with placental parasitemia, being born with placental parasite density between 1,000–4,999 parasites/*μ*L, being born with placental parasite density ≥10,000 parasites/*μ*L, and being born in a first pregnancy were all associated with the presence of umbilical cord blood parasitemia. Low birth weight was not associated with umbilical cord blood parasitemia (Table 3). 

Low maternal peripheral and placental parasitemia, prematurity, use of impregnated bednets, low birth weight, and female sex were independent risk factors for infection in the babies.

On multivariate logistic regression, none of the seven factors that were significant on univariate logistic regression remained significant (Table 4). 

## 4. Discussion

### 4.1. Low Levels of Umbilical Cord Parasitemia

This study shows the burden of malaria in pregnant women at the delivery unit in a malaria-endemic area of Burkina Faso. This investigation demonstrated the burden of malaria during pregnancy. The maternal peripheral parasitemia rate found in this study was 28.2%. This is similar to the rate reported in other countries in sub-Saharan Africa [2, 21]. 

The prevalence of placental parasites was 19.5%. Previously reported prevalence rates of placental parasitemia in Africa have been highly variable, ranging from 17.2% to 57% [11, 22–24]. The placenta is a site for *Plasmodium falciparum *sequestration. Many hypotheses, based on a systemic or local failure of the immunological response to malaria, have been proposed to explain the preference of the parasites for replication in the placenta [25].

The malaria rate in umbilical cord blood was low in the study population, occurring in 1.4% of all newborns, in 5.2% of babies born from mothers with peripheral and placental malaria infection, and in 5.1% of babies born from women with placental malaria infection only. Previously reported rates from different parts of Africa are highly variable, ranging from 0% to 54% [11, 22, 24, 26, 27]. The low rate in the present study, which is comparable to rates reported in some previous manuscripts, could be explained by effective prevention of malaria during pregnancy. In fact, it has now been proven that malaria parasites identified in cord blood are acquired antenatally by transplacental transmission of infected erythrocytes. The mechanisms underlying congenital transmission, according to an earlier report, include maternal transfusion into the fetal circulation either at the time of delivery or during pregnancy, through direct penetration through the chorionic villi, or through premature separation of the placenta [28, 29]. Pregnant women, who have received antimalarial treatment, should be able to clear parasitemia to avoid umbilical cord transmission. Immunity in the mother could also provide an explanation. Transplacental transmission of malaria appears to occur infrequently; it may be possible that after transplacental transmission, some elements of immunity, acquired from the mother, protect the infants from becoming infected [24]. This resistance may reflect, among other things, the physical barrier of the placenta to infected red cells, the passive transfer of maternal antibodies, and/or the poor environment afforded by fetal erythrocytes for plasmodial replication, due to their fetal hemoglobin composition and low free-oxygen tension [28]. 

Some studies have found high rates of transplacental malaria infection. The difference between those studies and our report could be explained by several factors. First, the high efficacy of malaria prophylaxis during this study may have allowed the women to clear their parasitaemia and therefore prevent placental and transplacental transmission of the infection to their babies. Second, our sole use of microscopy may have led to underdiagnosis, since this method has lower sensitivity than the newer molecular methods, such as real-time PCR, used in other studies [22, 29]. Finally, it might be that our numbers reflect the true prevalence of infection, as compared with data collected during routine services, in which lack of quality control of the reading of smears could lead to an overestimation of the infection rate.

### 4.2. Risk Factors

Univariate analysis of the association of risk factors with the presence or absence of umbilical cord parasitemia revealed seven risk factors that was associated with *Plasmodium falciparum* parasitemia in umbilical cord blood. The presence of maternal peripheral parasitemia, placental parasitemia, high-density peripheral parasitemia, high-density placental parasitemia, and primigravid status was associated with an increased risk of malaria parasitemia in cord blood. However, in multivariate analysis, these associations failed to reach statistical significance. 

Maternal peripheral blood parasitemia was associated with umbilical cord blood parasitemia (OR = 9.24, *P* < 0.001). Four cases of umbilical cord blood parasitemia occurred in pregnancies not complicated by maternal peripheral malaria infection. 

There were also associations between placental and cord blood parasitemia (OR = 10.74, *P* < 0.001), and between cord blood/placental and maternal peripheral parasitemia. Five cases of umbilical cord blood parasitemia occurred in pregnancies not complicated by placental malaria infection. Only two newborns had mothers with neither peripheral malaria infection nor placental malaria infection. Earlier studies of transplacental malaria reported these associations [11, 18, 24, 30]. A strong association between placental malaria and umbilical cord blood parasitemia has been reported, and this was suggested to be responsible for congenital malaria. However, whether the presence of *Plasmodium falciparum *malaria parasites in umbilical cord blood denotes infection acquired antenatally or contamination with infected maternal blood at delivery is not clear [15]. These associations suggest that clearing maternal and placental parasitemia with effective antimalarial drugs before delivery could prevent transplacental transmission of parasitemia. 

The density of maternal peripheral parasitemia (>5,000 parasites/*μ*L) was an important determinant of the likelihood of umbilical cord blood parasitemia (OR = 5.29, *P* = 0.004). The density of placental parasitemia (>10,000 parasites/*μ*L) was also an important determinant of the likelihood of umbilical cord parasitemia (OR = 4.91, *P* = 0.03). High density parasitemia seems to facilitate umbilical cord infection. Conversely, previous reports, showing low rates of transplacental transmission of malaria, suggest that the placental barrier is very effective in the case of very low malaria parasite density [22]. 

Finally, univariate analysis showed that being born from a first pregnancy was also linked to umbilical cord blood parasitemia (OR = 2.96, *P* = 0.02). 

## 5. Conclusions

Our data indicate that the rate of mother-to-child transmission of malaria, defined as positive umbilical cord blood parasitemia, appears to be low. Clinicians are therefore advised to investigate other aetiologies of fever in neonates. The low level of parasitaemia in cord blood suggests that contamination probably occurs during the labour period. Maternal, placental, and high density parasitemia were all associated with umbilical cord parasitemia. Prevention of malaria during pregnancy with effective antimalarial drugs should reduce the risk of infection for newborns.

## Figures and Tables

**Table 1:** Characteristics of enrolled women.

	Gravidity			
Characteristics	1	2	>2	All
Age, median (years)	19.24 ± 2.25	21.68 ± 2.60	28.71 ± 5.38	23.90 ± 5.89
Mean gestational age (weeks)	38.16 ± 4.24	38.79 ± 3.79	39.02 ± 3.59	38.66 ± 3.89
Mossi ethnic group (%)	95.8	96.2	94.7	95.5
Sleeps under bednets (%)	22.8	28.1	19.4	22.4
Mother death (*N*)	2	0	0	0

Pregnancy outcome				
Stillbirth (*N*)	19	8	23	50
Miscarriage (*N*)	8	3	7	18
Weight (g)	2,719 ± 430	2,893 ± 390	2,995 ± 440	2,875 ± 450

Number of live babies (*N*)	460	270	579	1309
LBW* (*N*/%)	111 (24.1%)	39 (14.4%)	57 (9.8%)	207 (15.8%)

LBW*: low birth weight.

**Table 2:** The proportion of newborns with *Plasmodium falciparum *parasitemia in umbilical cord blood, placental parasitemia, and maternal parasitemia, along with parasite density.

	Gravidity			
	1	2	>2	All
Peripheral parasitemia (%)	34.3	35.2	20.3	28.2
Geometric mean (parasites/*μ*L) (CI 95%)	2,143.18 (1,552.86–2,957.92)	894.71 (548.60–1,459.19)	495.09 (318.44–769.74)	1,106.47 (871.58–1404.67)
Negative peripheral parasitemia (%)	32.4	18.8	48.8	71.7
Peripheral parasitemia 1–999 (%)	30.9	29.5	39.6	11.4
Peripheral parasitemia 1,000–4,999 (%)	53.3	19.6	27.2	7.1
Peripheral parasitemia 5,000–9,999 (%)	52.5	22.5	25.0	3.1
Peripheral parasitemia ≥10,000 (%)	58.8	25.0	16.2	5.2

Placental parasitemia (%)	28.5	21.9	11.4	19.5
Geometric mean (parasites/*μ*L) (CI 95%)	1384.55 (880.97–2,176.00)	396 (223.30–703.17)	591.71 (289.60–1,209.00)	830.52 (599.66–1,150.27)
Negative placental parasitemia (%)	30.9	20.0	49.1	19.9
Placental parasitemia 1–999 (%)	46.5	26.4	27.1	10.1
Placental parasitemia 1,000–4,999 (%)	51.2	26.8	22.0	3.2
Placental parasitemia 5,000–9,999 (%)	50.0	29.2	20.8	1.9
Placental parasitemia ≥10,000 (%)	69.7	6.1	24.2	2.6

Umbilical cord parasitemia (%)	2.4	1.5	0.5	1.4
Geometric mean (parasites/*μ*L) (CI 95%)	263.74 (40.85–1,702.50)	644.08 (5.85–70,803.72)	222.16 (0.76–64,635.57)	315.69 (87.02–1,145.20)
Negative umbilical cord parasitemia (%)	34.9	20.5	44.7	98.6
Umbilical cord parasitemia 1–999 (%)	61.5	15.4	23.1	1.0
Umbilical cord parasitemia 1,000–4,999 (%)	0.0	100	0.0	0.1
Umbilical cord parasitemia 5,000–9,999 (%)	0.0	0.0	0.0	0.0
Umbilical cord parasitemia ≥10,000 (%)	66.7	33.3	0.0	0.2

**Table 3:** Univariate logistic regression of risk factors associated with *Plasmodium falciparum *parasitemia in umbilical cord blood.

	Proportion of newborns with umbilical cord blood parasitemia and the risk factor specified	Odds ratio	95% confidence intervals	*P*
Peripheral positive parasitemia	77.8%	9.24	3.02–28.28	<0.001
Peripheral parasitemia 1–999	22.2%	2.23	0.75–7.20	0.13
Peripheral parasitemia 1,000–4,999	11.1%	1.64	0.37–7.28	0.50
Peripheral parasitemia 5,000–9,999	22.2%	9.62	3.01–30.69	<0.001
Peripheral parasitemia ≥10,000	22.2%	5.29	1.69–16.52	0.004
Placental positive parasitemia	72.2%	10.74	3.79–30.42	<0.001
Placental parasitemia 1–999	16.7%	1.78	0.50–6.24	0.36
Placental parasitemia 1,000–4,999	22.2%	9.62	3.01–30.69	<0.001
Placental parasitemia 5,000–9,999	5.6%	3.13	0.40–24.56	0.27
Placental parasitemia ≥10,000	11.1%	4.91	1.08–22.28	0.03
Primigravid	61.1%	2.96	1.13–7.69	0.02
Prematurity (<37 weeks)	33.3%	1.58	0.52–4.25	0.36
ITN	33.3%	1.69	0.63–4.56	0.29
LBW*	27.8%	2.10	0.74–5.97	0.16
Female sex of infant	61.1%	1.78	0.68–4.63	0.33

LBW*: low birth weight.

**Table 4:** Multivariate logistic regression of risk factors, associated with *Plasmodium falciparum *parasitemia in umbilical cord blood.

	Odds ratio	95% confidence intervals	*P*
Peripheral positive parasitemia	2.43	0.50–11.66	0.26
Peripheral parasitemia 5,000–9,999	3.71	0.92–14.98	0.06
Peripheral parasitemia ≥10,000	1.60	0.38–6.66	0.51
Placental positive parasitemia	3.30	0.73–14.87	0.11
Placental parasitemia 1,000–4,999	2.27	0.58–8.77	0.23
Placental parasitemia ≥10,000	0.95	0.15–5.69	0.95
Primigravid	1.91	0.70–5.21	0.20
